# Clown’s view as *respiciō*: looking respectfully to and after people with dementia

**DOI:** 10.1007/s11019-016-9734-1

**Published:** 2016-09-23

**Authors:** Ruud Hendriks

**Affiliations:** Department of Philosophy, Faculty of Arts and Social Sciences, Maastricht University, PO Box 616, 6200 MD Maastricht, The Netherlands

**Keywords:** Elder-clowning, Embodiment, Care ethics, Person-centered dementia care, Reciprocity, Respect, Sense perception

## Abstract

Clowns seem suspect when it comes to respect. The combination of clowning and people with dementia may seem especially suspicious. In this argument, I take potential concerns about clowning in dementia care as an opportunity to explore the meaning of a respectful approach of people with dementia. Our word ‘respect’ is derived from the Latin *respiciō*, meaning ‘looking back’ or ‘seeing again’, as well as ‘looking after’ or ‘having regard’ for someone or something. I build upon this double meaning of *respiciō* by examining how simultaneously we look to and after people with dementia. I do so empirically by studying how miMakkus clowns in their practice learn to look with new eyes to people and things around them. I call this clown’s view and differentiate it from the predominant way of observing people in dementia care. I argue that *respiciō* comes in two guises, each of which merges specific forms of looking to and looking after the other. By making conventional, solidified ways of seeing the other fluid again, clowns remind us of the value that comes with a veiled way of paying respect to people with dementia.

**Respicere**

1. *Present active infinitive* of respiciō

**Respiciō**

From *re-* (“back; again”) + *speciō* (“observe, look at”).

1. I look behind, look back at or upon, look to, look around.
2. I have regard for, consider; respect.

Our seeing is not pure visual perception. Our seeing implies intentions and meanings and as such it is an expression of our lived relation to our environment. …Ways of seeing are ways of being… : how as observing beings do we relate to our environment and to each other? (Kamphof 2012: 13)

## Introduction

People often react slightly amazed when I tell them about my research on miMakkus, a special method of clowning for people living with an advanced stage of dementia. Most of us are likely to associate clowns with children rather than the elderly, let alone seniors with dementia. The colorful character of the clown contrasts sharply with the sterile image of nursing home care. A clown’s expressive vibrancy epitomizes vitality, and this seems at odds with the routines of everyday life in the psycho-geriatric ward (PG). Commenting on the two worlds, one expert said that “the paradox could hardly be greater.”^1^

The culture of dementia care is undergoing changes, however (Griffin 2012). Today’s vision of quality care stresses at least three elements: it is person-centered, it engages people in meaningful activities, and it promotes their connection with the outside world (Kitwood and Benson 2004). More or less creative day activities therefore satisfy a major need. Professional artists, too, increasingly find their way to the PG. Where conventional communication fails, *art* might well succeed in reaching the person with dementia (de Medeiros and Basting 2013).

Similar expectations about the power of art apply to the specialized forms of clowning that in the past decade emerged in intramural care for seniors (e.g. Raviv 2013; Symons 2012). Elder-clowning evolved in the wake of other creative approaches aimed at supporting vulnerable seniors, improving their quality of life, and countering the threat of loss of identity (Basting 2009; Lee and Adams 2011; Warren 2008).

The goal of miMakkus is to make *contact* with people who are not (any longer) capable of communication in the usual, cognitive manner. The so-called ‘miMakker’ (‘my pal’) is not an entertainer who rehearses an act, performs a trick, or tries to make people laugh. The miMakker is concerned with the emotional wellbeing of residents, their experience of “joy and sorrow” (www.mimakkus.nl). A miMakker focuses on the individual and tries to go along in how this person experiences a situation through improvising in a subdued, non-theatrical way in response to what takes place in the here and now. She thereby makes contact beyond ordinary language and meanings.

“How special,” as many people reacted when I told them about miMakkus. “Not for me,” as others whispered who saw it as a matter of little *respect* to send a clown to people who cannot rationally assent or object to it. It involves vulnerable patients, they reasoned, who can be easily misled.^2^

Respect is a thorny issue in public debates on care for people with dementia. One context in which the notion of respect appears is that of living wills and their status. This pertains to respecting the will of the person who when still capable of rational decisions indicated a desire for not wanting to live any longer in the event their suffering from dementia becomes unbearable. The suffering feared is closely interconnected with the fear for the loss of personal identity and human dignity and, after having fallen prey to dementia, for becoming fully dependent on others.

Discussions on respect for people with dementia will soon center on big notions such as autonomy, identity, and dignity.^3^ In contrast, I am more concerned with the ways in which respect takes shape in small, everyday interactions with residents of nursing homes. The sense of discomfort sometimes prompted by miMakkus suggests, however, that weightier interests are at play. I consider this discomfort as a welcome occasion for further reflection on respectful dealing with people with dementia.

Respect comes from the Latin *respiciō*—the verb being *respicere*—which means ‘looking back’ or ‘looking again,’ in addition to ‘looking after,’ ‘being concerned with’ or ‘caring for something or someone.’ In my argument I capitalize on this double meaning of *respiciō* by exploring how we look to and look after people with dementia. I do so empirically, by investigating how the miMakker learns to see with other eyes (‘clown’s view’) and how this differs from common ways of looking at or dealing with people with dementia in care. The question of how clown’s view respects the other is center-stage.

In the context of this research project I enrolled in the special training course for miMakkus clowns. This training, which I successfully completed in March 2009, comprised 22 days of instruction, field visits and an internship trajectory. Based on participant observations I made field notes. During a second round of ethnographic fieldwork I followed five miMakkers at work for a week (July 2010; January 2011; February–March 2011; May–July 2011). Finally, I observed four miMakkers-in-training (November–December 2011) during one of their hands-on internships.^4^

In my argument below I briefly discuss the background of the discomfort sometimes triggered by clowns. I follow one miMakker in her practice to understand the stakes and power of clowning. I address several contentious aspects of clowning to trace back the difference in valuation to the two forms of *respiciō* distinguishable in dementia care. Finally, in my conclusion I draw up the balance regarding the specific contribution of ‘clown’s view’ to a respectful treatment of people with dementia.

## Disruption as counterbalance

Who plays the clown calls suspicion on herself when it comes to respect. With her red nose, which provides her with an alibi “to look at the world differently,”^5^ the miMakker situates herself in a cultural tradition harking back to the fool—a figure in the margins of authority who without fear of being of punished for it could mock the pretention of power. As *other*, the forerunners of modern clowns functioned as critical counterpart of the established order and it was their role “to re-establish an equilibrium in the face of power, which is immobile and static” (Robb 2007: 6).

Simonds and Warren (2004) have demonstrated how precisely a modern hospital will benefit from a figure who enjoys special protection to disrupt the order. Once a relation of trust has been forged, hospital clowns are allowed to do anything in response to patients and staff. “They push the limits. In so doing, they shake up the hierarchy and protocols and indirectly serve the healing process” (64). The power of the clown resides in a playful reversal of roles. Regardless of their knowledge and authority, the clown exposes the care-provider as victim. Patients—such as children who have to undergo heavy treatment—deserve her respect. With the clown as ally these patients are allowed to be in charge, if only for a moment.

As a distant descendant of the fool, the miMakker, too, is free to disrupt the prevailing morality or to cross boundaries. Take Fien, the clown character of Tessa Brouwer (a pseudonym) and one of over two hundred miMakkers who successfully completed their training.^6^ A miMakker puts much of herself into her clown, which is why these clowns differ as much in character as in appearance. Fien can be characterized as a French *artiste*, the Edith Piaf type. As such she represents many shades, but as no other Fien embodies the rebellious side of clowns.

Fien is keen on fooling authority figures. An unsuspecting care-provider who had to sneeze loudly, for example, immediately faced the echo of a strongly exaggerated clown’s sneeze. Commonly, such spontaneous reactions are not meant to criticize care-providers—the clown is primarily geared to *residents*: with a secretive look towards them Fien still tried to bury her sneeze in the net curtains. There certainly are good reasons to hold the mirror up to care-providers every now and then, but the miMakker primarily aims to create a sense of togetherness with the resident.

## Clowning contested

A clown can take the liberty to do things that others cannot. The clowning approach thus turns prevailing power relations upside down, but also tampers with convictions of what is ‘good’ in care for people with dementia. Take Fien, whose loose shoelaces are a harbinger of the trouble she constantly gets mixed up in. A conscious strategy: the clown’s clumsiness has an irresistible appeal for those who commonly find themselves in a vulnerable position and who are thus offered a chance to take care of her (cf. Hendriks 2012). A first field note:

*Saint Anna, 22 February 2011, 10.05* *h*

“Watch out! Or you fall down!,” as the residents warn Fien to be cautious. Fien hesitates. “Just walk on,” as the women concernedly say to her. “Walk carefully, as you always do.” “Like this?,” Fien asks. “Yes, go ahead!”

Of course she does fall down, stretched out to the floor. Although some will find it inappropriate for a grown-up woman with a clown’s nose to be mopping the floor, most skeptics will accept its reality. As long as their mom or dad just sit there quietly, and do not have to do anything… But once Fien finds herself stretched out on the floor other forces come into play.

*Saint Anna, 22 February 2011, 10.35* *h*

Mrs. De Groot motions Fien, who goes to her by crawling across the floor. Fien finds a stool to sit on, but it still needs to be polished: she is quite a “clean person,” Fien says. Her gaze falls onto the crust of bread lying on the floor. She picks it up and shows her find to Mrs. De Groot, who grabs the crust and puts it into her mouth. A tiny bit remains in her hand and Fien puts it into her mouth without a blush. Carefully chewing on it she tastes it. A moment later they are chewing together, heartily. In the meantime a conversation develops between them that consists of soft sounds accompanied by changing facial expressions. They completely understand each other, so their body language reveals, even if ‘oops’ and ‘wow’ are the only words I can make out.

The discomfort potentially triggered by this situation has first of all a personal dimension. Close relatives of dementia patients often do their utmost to affirm the identity of one who can no longer do so himself by holding onto his earlier values, preferences, and habits. Kitwood (1997: 20): “The more that is known about any individual’s interests, tastes and life history, the more likely it is that this need will be met.”^7^ Unlike relatives, friends, and care-providers, Fien as quasi-naïve outsider lacks such shared history with the resident. Although before visiting a home miMakkers inform themselves about possible clinical or social details, and, depending on the frequency of visits, get to know ‘their’ residents over time, such information should not be allowed to get in the way of their clown’s play. MiMakkus training devotes much attention to techniques (more on this later) to look at the other with *new eyes* time and again.

It is a question, however, whether close relatives of Mrs. De Groot can still see the person she *was* in the one who shares a crust of bread with Fien, and if she herself would have wanted to. By focusing on the here and now the personal history of the other threatens to vanish from sight.^8^

Secondly, the clown’s nearsightedness leads to a tension with the conventional outlook on humankind hidden in views about and practices of good care. Arno Huijbers, initiator and instructor of the miMakkus method, suggests that in the early stages of dementia patients may find a hold in references to the familiar reality, but that for people in later stages it has a crippling effect when they are constantly corrected because of their experience being at odds with (our view of) reality. The clown precisely tries to reach out to this last group. She wonders whether she can *go along* in, and possibly add in an imaginative and playful way to the other’s language and experience.^9^

But this can also be perceived as debasing. Should you go along in the loss of decorum that, as some argue, occurs in people with dementia? To prevent the other from sinking even further, we commonly try to shelter him or her in the order of meaningfulness as long as possible. All and everything has its own place in it: people at the table, food on the plate, dirt on the ground.^10^ Who like Fien deliberately disturbs this order does not only ignore the kind of person Mrs. De Groot *was* before she lost her language and memory; potentially one also undermines the dignity of the person the other still *is*.

## *Respiciō* from overview

Ideas on what is ‘good’ in contact with people with dementia, and how to evaluate the miMakker in this regard, thus turn out to diverge strongly. To interpret this difference in assessment we need to go back to the origin of the term ‘respect’: Those who elevate the notion of going along in the vulnerable position of people with dementia to the art of clowning have a *way of looking* that differs from those who fear that people’s sense of dignity may thus be adversely affected. This difference goes back to two guises in which *respiciō* becomes manifest in dementia care, which represent two traditions in the history of the Western gaze. I derive them from a study by philosopher Ike Kamphof, entitled *Iedereen voyeur* (Everyone a voyeur, 2012), in which she describes the birth of the modern control-oriented gaze (overview, oversight, surveillance) as well as an older form of ‘nearsightedness’ that still colors our vision.

Kamphof starts off her history of the modern gaze with the early Renaissance author Petrarch who, having climbed to the top of *Mont Ventoux*, reported on the visible world at his feet, but he was also—and this was a first—fully “aware” of the *experience* of having a (panoramic) overview (22). Many would follow in his footsteps, turning the “entire earth into a domain of observation” (23) and mapping the unknown. Kamphof writes about a “newly acquired inner footing” that offers the heirs of Petrarch “a hold” (23). “From that perspective, they view themselves as autonomous beings, separate from and partly placed above nature” (23). In this way they acquire “knowledge about the world” (23) and a certain preponderance regarding what they look out upon from their lofty position. The articulation of an objectified worldview and a modern subject perceiving it as such go hand in hand.^11^

By appropriating the world as visual object, the observing, understanding human being becomes a subject. No longer is he a small element in a divine creation, a toy of fate and nature. He is outside and above what he understands by observing. The world becomes his world. (23)

Given the omnipresence of this way of looking we are hardly aware of it anymore. Modern patient care is no longer conceivable without detailed observations and “scientific objectification” (28) that within the logic of evidence-based care counts as decisive good.^12^ Statistical data processing and schematic representation of observations on an aggregated level thereby mirror the “distance of the spectator to his image” (39–40), which is characteristic of this meanwhile conventional way of looking. But observation also has an everyday dimension. The miMakker regularly engages with her work as an observer as well. This applies for instance during her training, when she observes the practice of experienced colleagues, as well as for the miMakker who observes and reports on the effect of her own interventions.^13^

## Overview as moral attitude

Through observation we learn to know the world in a certain way. Our way of looking, however, is not only of importance epistemologically, but also has normative value. The double meaning of *respiciō* reminds us that our relation to the world is expressed not only in how we look *to* the world and ourselves, but also in how we look *after* the world and each other—people with dementia in this case.

The conventional modern perspective is ruled by distance (or detachment) in normative respects as well; it calls for appropriate distance, both between individuals and vis-à-vis the world. In the wake of this modern way of looking, respect primarily came to refer to the *autonomy* of the subject. Translated to ‘good’ care for people with dementia, all is geared maximally to underpin the ‘grand’ subject position they once shared.

There are ample threats, however, in the form of loss of language and memory and reduced awareness of reality. Dworkin (1993), a prominent representative of this normative perspective, argued that people suffering from advanced dementia “have no sense of a whole life, a past joined to a future, that could be the object of any evaluation or concern as a whole” (230). Precisely the competence of these people to *survey* their life as an *integrated totality* and use this as a basis for reasoning, as Dworkin claims, is undermined by serious dementia. As a result, they have “no contemporary opinion about their own critical interests” (230).

Resisting biological destiny, this form of respect is aimed at helping patients to retain their inner footing. The threat of loss of control or overview must be compensated by making life more ‘surveyable’. A respectful approach contributes to memorizing and understanding, offers knowledge and power by supporting the act of choosing. Informed consent is the normative rule. If this is not possible anymore and we hold on to the consequent application of the autonomy principle, in line with Dworkin’s argument, the person’s earlier set of values—from before dementia gained the upper hand—deserves our respect (cf. Jaworska 1999: 108).

This conventional way of looking offers a moral compass in dementia care, providing arguments against an exclusive focus on the quality of the momentary experience that we, playing the devil’s advocate, might suspect the miMakker to have.^14^ Or, in Dworkin’s terms: who concentrates on fulfilling experiential interests will be more inclined to lose sight of a person’s lasting values (or critical interests)—that is, concerns about whether something will fit in with a person’s course of life, with what he or she always believed to be right (cf. Jaworska 1999: 108–109). A conventional way of seeing and being fosters and explains skepticism about the role of the clown, who in her focus on the *now*-*self* of the resident would forget the *then*-*self* (cf. Koppelman 2002).

This said, it is not as if the miMakker never doubts whether she is right or would be insensitive as regards to the inviting perspective of control. In how she looks after or cares for the other, the clown also draws from this repertoire of *respiciō*, which considers the autonomous subject as standard. During their training, miMakkers are told to “always ask for permission,” for instance to sit with someone—they need to deal respectfully with desires and the privacy of residents. “Coercion is altogether wrong,” as Jan Rauh (2013), one of the first miMakkus instructors, claims in his personal notes: “When a resident does not *want* any contact, we will *respect* this and go away” (34, emphasis added).

### *Respiciō* from receptiveness

Sometimes the desire of a resident and the question of how to respect it leave little to guess. “Just you go and knock on the next door!” I was told as Joop—my alter-ego as miMakker—during one of my first internships. Others may like it but I do not—this is how a clown may subtly be put in his place. Commonly, however, life in the PG is less transparent. Often it is hard to know what someone means because ‘normal’ communication is hampered and residents may not be able anymore to look at the totality of their own life. In the practice of care for people with severe dementia, the form of *respiciō* that centers on the autonomous subject proves to be all but taken for granted.

This is not to say that this way of looking would (or should) not play a role in care for people with dementia. It does in fact, as normative ideal—we usually continue to approach the other as autonomous subject as long as possible—and perhaps as shared reality as well. But as Kamphof (2012) writes, ‘overview’ and control also come at a price because they cover an alternative way of seeing and being (53). The modern role of the observer-at-a-distance was preceded by another way of looking, which—discreetly, “as substratum of our perception” (47)—co-constructs our relation to the world and the other. This other form of looking and looking after—*respiciō*—starts from “our receptiveness for that reality” (37).

In what follows I discuss this alternative way of looking on the basis of Kamphof’s remarks on visual culture.^15^ Next, I apply it to the gaze of the miMakker (‘clown’s view’) as developed in contact with residents. I will extensively address how clown’s view emerges in actual practice, before I finally elaborate the normative implications of a receptive way of looking for the encounter between the miMakker and the resident.

Kamphof traces back the origin of this alternative way of seeing and being to the dusty attics of art history, where particularly religious images and artifacts seem to cross out the prevailing view in art philosophy that there is a clear dividing line between image and reality. In the visual culture in which believers lovingly touch their icons and images—or where, conversely, such images provoke anger—there is no “observer who keeps a distance”; rather, looking includes “forms of direct sensory and emotional response” (42).

From a modern perspective such intense dealing with images may seem archaic. And yet, many of us are also easily “tempted” by images (42). An image of a person or event may offer us—through its confusing similarity with the original or by having been physically in touch with it—a “magical” experience as well, one of “coming nearer to otherness” (44). Looking makes the outside world resonate within us. You do not experience a “deeply touching” movie as observer; rather, such movie “immerses you in the reality presented” (45). Looking is something we do with our entire body, as Kamphof writes with reference to Sobchack: “Seeing is also touching, smelling, hearing and feeling a body move through the space in front of us” (76).

The *inner* footing that gave the modern subject a sense of autonomy and control is accompanied, according to this alternative history of the gaze, by a *physical* footing of seeing in the real world, and this we also find with the clown. The ‘self’ depends on that world for sharpening the senses. “A single matted color leaves our eyes blind” (47); a lack of contrast puts out the senses.

Sometimes this overlooked foundation of our perception briefly reveals itself. Kamphof indicates how modern visual art commits “an attack on our gaze” that causes our view of the world to vanish, and with it “the certainties on which the self is founded” (48). The artwork knocks the observer off his pedestal. “We lose our place as beholders of a well-shaped and understood image. In a way we become blind for the modern artwork” (48).

My point is not to make a case for visual art as such, but for what it reminds us of—our half-forgotten power “to see again, this time with other eyes, sensing new possibilities” (48). Art teaches us to distinguish new nuances in what first seemed chaos or left us indifferent, and to let ourselves be touched. In such a moment the “boundaries between self and world” get blurred and for a moment we feel that we owe our ability to ‘see’ to the “perceivability of the world” (49). On such moment it is “as if we received eyes only a minute ago” (47), Kamphof writes with reference to Lyotard. Momentarily the eye becomes “an organ for touching” while seeing is briefly “being touched” (49).

## Clown’s view

A miMakker, I argue, embodies this often overlooked, receptive form of *respiciō* as no one else. She is one who looks in an engaged manner, by using her entire body: becoming a miMakker can be understood, as I also argued elsewhere, as *acquiring a sensitive body* that in ever greater detail learns to distinguish between subtle signals and differences in how the other is present in the world in an attentive, physical and sensorial way (Hendriks 2012: 469).

Her sensitive clown’s body helps the miMakker to attune to the other “like an antenna.”^16^ “Fine-tuning” is a matter of “searching for the other’s wavelength, wherever that may be”—not by observing the person with dementia from a distance but by trying to “go along” in his emotion, physical presence, sphere of attention, and experience. By adapting to the rhythm of breathing and a slower tempo, for instance, “you respect the resident.”

As practice of *mimesis* (Taussig 1993), this clowning technique escapes the contradiction of (mental) immersion versus (strictly outer) imitation. By playing along, the miMakker tries to *incorporate* the world of the other as a way to approach the other’s experience. “I am not going to copy or imitate someone,” as Jan Rauh teaches us, but:

Lesson 1 *‘Presence’, Eindhoven, 12 March 2008*

JR: The moment that you [as resident] walk like this, I as clown can join in; … I then take over that *disposition*. In this way you [as clown] can sense how it could be for me [as resident], and next you may hook up with that and perhaps we thus find something we can share.^17^

Attunement is a challenge and calls for commitment; it requires “courage” to let yourself be carried along by the disorderly situation you and the resident may end up in. Precisely by relinquishing all certainty, shared contact may emerge—beyond the autonomous subject, “as if it happens *to* you.”

Such a moment of being together can potentially enrich the life of residents, as I will argue below—for the miMakker it is a vital gift. “Being open to outside stimuli” is at the core of who she is: Fien would never have become the sensitive clown she is without the subtle shades that *residents taught her* to see. By explicitly expressing her acknowledgment to the resident, Tessa Brouwer always recognizes what she owes to the other: the articulation of her clown’s view.^18^

The clown must put in an effort, however, to acquire the receptive habitus that allows the world to resonate in her. It takes a lot of exercise to tap this sublayer of perception and to learn to use it for making contact (Hendriks 2012: 469–70). A first step is to “empty” yourself. In our training we were offered various tactics to “rid ourselves of meaning,” as in an exercise in which we had to explore the material space and things “through the eyes of a child” for whom all is “totally new.” Through coaching and various exercises, the miMakker is trained to move away from the familiar way of looking and explore new possibilities with other eyes—to come out of the dark and see the world in new ways.

Internship 1 *Evaluation*, *Maastricht, 11 September 2008*

TS: You open your suitcase and say “Shall we have a look.” No, go along with her, for you do not know either what is in that suitcase. With every new moment, you as clown are new as well; it is also a matter of your sense of wonder! We simply think intuitively… suitcase: something is in it. No, the suitcase is a secret as well.

The clown does not know what she sees.

Lack of overview causes certainties on which the self relies to disappear. But instead of holding to an *inner* footing and use it as base for looking at the world, as I was inclined to do with my suitcase according to my internship supervisor Trudy Schambergen (at the time training director at miMakkus), the clown is taught to find a *physical* footing in the world. It is called “grounding” in theater terms, for instance by shifting your attention from your head full of ideas and thoughts to the weight of your body and the floor that “carries” you. The miMakkus clown Pip, for example, owed her new sensitive self to her surrender to the floor beneath her feet, that with her powerlessly probing clown’s gaze she saw transform into a frighteningly thin layer of ice. Thus she learned *to forget* what she as autonomous subject *knew* about the floor, when she still was simply Patricia and the floor still was a well-grounded object outside of her (Hendriks 2012: 464).

Striving for equal contact requires not only letting go of certainties and connecting to the other, but it also implies that the miMakker can be *touched*—affected, moved, stirred—by the other.^19^ Clown’s view includes forms of sensorial and emotional response that in case of contact may overwhelm the miMakker with joy, but as clown she may also be hurt by the other. “You open up and therefore you can be moved any time.” Without protective layer of language and other resources to protect herself against the outside world—as sensitive body—the clown who is fully present is also defenseless in the world. This also implies that Fien, precisely when from a modern perspective she goes too far and threatens to lose her respect for the other, in her own eyes is at her most vulnerable and hence preeminently respectful.

## Clown’s view as moral attitude

The normative implications of clown’s view strongly differ from those of *respiciō* in its modern guise (cf. Overview as moral attitude). A respectful clown’s gaze is ruled by *proximity* rather than detachment; it calls for seeking proper rapprochement. The clown-like mode of looking after is no longer aimed at preserving autonomy; its primary concern pertains to the individual as relational and embodied subject. The clown represents this role in optima forma herself, but the underlying subject view pertains to each and every other. Translated into a proper approach of people with dementia, the miMakker considers it to be a matter of respect to try and go along in the ways in which the other is present in the world and to optimally support and secure their connection to the world.

Again, dementia comes with many threats for the relational and embodied self: a changing bodily experience, limitations in sensorial perception and processing of stimuli, hidden pain, uncertainty, fear, and depression. These signs and symptoms are often reinforced by negative influences from outside, such as a monotonous living environment designed without an eye for beauty, absence of attention and touching, lack of sense of security, and so on.^20^ They potentially undermine precisely the already vulnerable ability of people with advanced dementia to remain *receptive* for external stimuli and to continue playing an active part in sensory conversations with the world.

In contrast, a respectful approach based on clown’s view is geared to helping people with dementia to retain what is being threatened: their involvement in the world. The miMakker does not only cultivate her own receptive gaze, accepting the residents’ subtle presence as a gift for the articulation of her clown’s view, but calls upon a similar, hidden layer in how the resident looks to and after the world that she as clown can help articulate. By being all but grey and instead fostering the senses the clown in her turn seeks to make a difference. She creates specific conditions that offer residents the opportunity to regain a sensitive body, not only to reach out and touch but also to undergo stimuli and let themselves be touched—in short: to see anew—in order to prolong their ability to experience their sensorial and emotional footing in the world. Clowning in dementia care is thus tantamount to creating conditions in which mutual articulation may occur, allowing both clown and resident to tackle indifference and see each other once again.

To underline the extent to which clown’s view as moral approach differs from a conventional modern way of respecting the other, the following sketch, in which all conditions for informed consent seem to have been met, is illustrative: Mrs. Vriends unmistakably rejects Fien’s invitation. But instead of taking that response for granted, Fien tries to find out if Mrs. Vriends perhaps *wants* contact but is unable to *realize* it. If someone does not want contact, I quoted Rauh (2013), the clown must respect it. “But if a resident does want to, but is unable to,” he continues, “we need to do our best to make contact” (34). This qualification implies the transition from a form of *respiciō* that prioritizes the autonomous will of the other to the alternative provided by the clown that centers on our emotional-sensorial connection with the world.

Rather than keeping a distance in the absence of overview and looking for sources that—as external memory and conscience—might provide knowledge about what the resident might have wanted (cf. Dworkin’s moral guideline), for instance by seeking consent by proxy, the miMakker leaves “the last word” up to the resident. The voice of Mrs. Vriends, however, is not unrelated to the context in which it can be articulated (cf. Pols 2005). The clown asks for agreement, so Rauh (2013), by means of attunement: “What does the resident want? Through optimal attunement, the miMakker needs to find out” (34). But attuning, he adds, “is hard because something can be different again in an hour” (34). Which is not to deny that the clown can try again and again to “see the other, touch the other.”^21^ *Respiciō* in its receptive form also means: modifying the conditions under which people see each other in a way that they may come out of it differently.

*Saint Anna, 1 March 2011, 10.35* *h*

Outside a bird is chirping. Fien whistles towards the bird and Mrs. Vriends whistles along with her. Fien reacts thrilled. She picks up her guitar and songbook. Mrs. Vriends says this is not allowed, at least she *believes* it to be so. “Oh no? Shall I call again?,” Fien asks ready to help. “Let it go,” Mrs. Vriends says. Fien picks up these words in her singing. “Let us go…,” she softly sings. “Miss!” Mrs. Vriends reacts in a plaintive tone. “Miss… can we gohoho,” Fien sings in more melancholy tone. “Just stop it,” Mrs. Vriends reacts after a while. Saddened Fien withdraws, but from some distance she asks for permission to go on through her gaze. She sings a song and another resident joins in. “Just quit it, it is enough,” Mrs. Vriends maintains. As a way to end, Fien passes the hat round. “No, just go away,” Mrs. Vriends says angrily. Fien takes in the rejection and reacts aggrieved. She magnifies her indignation by grumbling, stretching the scene’s ending. “Bye?!” she says with emphasis, almost as a question. “Just go,” Mrs. Vriends responds. Fien grins and whistling she gets her stuff out of the way. And still greets her: “Bye, bye!”

If we were trained at miMakkus how to deal with rejecting reactions from residents, we were told not to take it personally. It was also said that “the clown always says yes” and “accepts the gift.” But I never quite understood how to do this in the case of rejection, although it certainly did not mean that you simply had to go away. After all, the clown “embraces the dilemma,” as it was called in jargon, and turns it into a play which potentially gives rise to a shared experience. Yet it was Tessa Brouwer who first taught me what it means to say “yes” in a respectful way to a “no”, and thus to embrace the moral dilemma that presented itself.

Where normally we would perhaps be inclined simply to accept the negative emotion of Mrs. Vriends—by looking or walking away, out of respect for her wish and also because we may have other things to worry about,—it is precisely a matter of respect, according to Brouwer, to “stay close” to how Mrs. Vriends is present and to explore her presence cautiously.

Attuning to the other is a matter of “a continuous search” and of “looking: what are you doing? Which language do you speak? Where does this person find herself, in which emotional sphere?” Is this woman perhaps apprehensive or depressive, do uneasiness and confusion play a role? And it means in particular: can I go along in (i.e. incorporate) her situation or reverse it? Can I make her discomfort “melt” or should I just be with her to optimally raise “chances of contact” for her?

## Conclusion

Fien’s search can be read as an attempt to rescue human contact from the burden put on it by language and cognition. She is prepared to go far, too far according to some, namely as far as sharing a crust of bread on the floor. As a miMakkus clown she renounces the mental and physical distance of the subject towards the world that is commonly seen as an achievement; in both a literal and figurative sense, her intimate contact with the floor seems hardly ‘elevating’ to the other. But do the conventional rules of respect apply to the situation that unfolds? How do we know if Fien—on the floor or elsewhere—sees it right?

Whether or not the miMakker approaches the other respectfully, according to *respiciō* in its modern guise, the degree in which the clown supports the perspective of the autonomous subject is decisive. This form of respect is one of proper distance between subject and object, as well as between subjects, which gained an established position in psycho-geriatric care through notions such as permission, empowerment, and free will, some of which can also be found in the clown’s baggage.

Sometimes the world of dementia care will look orderly. But more often it won’t, which means that one should draw on another repertoire in order to sustain a respectful approach of the other. The miMakker practices an alternative form of *respiciō* that has the relational subject as yardstick and that taps everyone’s latent ability to stay involved in the world—even in an advanced stage of dementia—through a mode of sensorial conversation.

Some tentative lessons can be drawn from clown’s view for an ethics of dementia care. Similar to conceptualizations of the human subject in care ethics (Tronto 1993; Winance 2010), the clown’s respect pertains, on the one hand, to people’s *shared vulnerability*—the latent capacity of residents and others alike to be captured and moved by the world and by others, and this is what their articulation as a subject depends on. On the other hand, clown’s view honors *people’s presence*—the latent capacity shared by residents and others to make a difference to others, as a gift that offers them an opportunity to regain their sensorial and emotional footing in the world and become articulated as a subject.

Such capacities may be intrinsic to people, actual *engagement* in reciprocal relations of care is “not an innate human capacity,” as Mol et al. (2010: 14) remark in reflecting on the logic of care. Learning to ‘see again’ (the term used in my analysis) is rather “infused with experience and expertise and depends on subtle skills” that require training and fine-tuning “along the way” (14). As regard questions of good care, whether a clown sees things right cannot be judged unequivocally “from the outside” in the abstract terms of rule ethics, but is a matter of “practical tinkering” and “attentive experimentation” (13) that is situated in the “complex ambivalence” (14) of everyday care situations that an ethic of care attends to. Prone to failure rather than a recipe for success, supporting and validating people’s engagement with the world implies a propensity to not give up trying in the eye of uncertain outcomes.

Looking after the other’s vulnerable and affective presence in the world is a vital element added by the clown to person-centered dementia care. But clown’s view cannot be unequivocally translated into a list of guidelines for others to implement. To what extent (aspects of) a clown’s receptive form of *respiciō* can be carried over or inspire other art- and care practitioners, and which conditions are the most favorable to seeing again, is an empirical question. Supporting people’s embodied relatedness to the world is best understood as a process of “meticulous joint exploration” (Winance 2010: 100) by all involved in the process of care. As it touches on the essence of care, it is our collective responsibility—and a duty of guardians of the autonomy principle in particular—to make room in conventional care settings to further explore, cultivate and critically evaluate the clown’s form of respect, to the benefit of all involved in these relations of care.

As regards to how dementia is commonly approached in the world of care and in society, the miMakker takes up a special position, because her effort is aimed at rendering solidified ways of looking at and looking after people with dementia fluid again. But every human being is different, as is true of every encounter. In what measure disconnected experiences of someone’s current self and critical interests of his earlier self can be forged into a whole and in what measure clown’s view contributes in concrete cases to “seeing whole” (Hughes et al. 2006: 4) can only be assessed by combining different ways of seeing (Schermer 2003: 79)—overview, which assumes knowledge of the earlier self of the other (e.g. by a close relative, cf. Taylor 2008), and clown’s view, which assumes a particular way of looking to and after the other with fresh eyes.

## Footnotes

1 Rob Spijkers is a psychologist (Orbis, Sittard, The Netherlands) who has worked in psycho-geriatric care for 14 years. Interview: Maastricht, 16 June 2009.

2 On ethical dilemmas around truth and deception in care for people with dementia, see Schermer (2007).

3 See for instance Hertogh et al. (2007) for a contribution to the debate on the status of living wills of people with dementia in which respect for autonomy is central. This debate builds on the work of Dworkin (1993), Dresser (1995), Jaworska (1999), Koppelman (2002), and others.

4 My observations were recorded within the framework of the project Beyond autonomy and language (The Netherlands Organisation for Health Research and Development, program Disability Studies in Nederland. See: www.zonmw.nl/en/). Prior to the project I received permission from all psycho-geriatric care facilities involved, as well as from the miMakkus Foundation in Eindhoven, the Netherlands, miMakkus instructors, and the (student and certified) miMakkers involved.

5 Arno Huibers, Lesson 3, *Body as tool*, Eindhoven 2 April 2008.

6 For privacy reasons I anonymized names of the miMakker clown-characters and practitioners, residents, and institutions, but not the names of miMakkus instructors. Given the proportion of women to men (of about 9–1) I prefer to use the feminine to refer to miMakkus clowns; where my own experiences are concerned, I use he/his/him.

7 Kitwood (1990) has pointed to the role of positive, social-psychological environmental factors—such as offering solace or a sense of comfort, confirming the reality of the other—that contribute to sustaining people’s sense of who they are. Negative factors are, for instance, exclusion, stigmatization, and denial of how someone experiences reality. According to this holistic vision on dementia, people who because of their vulnerable condition are frequently confronted with their failure or incapacity would experience much additional adverse impact of a negative social-psychological environment.

8 I should specify here that to win support miMakkers increasingly organize information meetings for relatives and others on the how and why of the work they do. The miMakkus-clown instructor Titia Brassé (Interview, Geleen, 10 April 2015) explains that the residents she visits as a miMakker need a formal indication for this type of care, to be specified in a care plan on which consensus is sought. She will not ignore a resident without formal indication who is looking for contact, Brassé says, but where possible one should also look for a dialog with relatives.

9 Arno Huibers, Lesson 21, ‘*Clown principles’*, Eindhoven 19 November, 2009. By embracing “the imaginary as a means to counteract barriers to communication such as residents’ incoherent or nonsensical speech in the presence of reminiscence or delusion,” as Kontos et al. (2015: 12) put it, the clown finds herself in the good company of other art interventions aimed at helping people to sustain a meaningful relation with their environment beyond cognition. For example, Anne Basting (2009), in the Time*Slips* project (http://www.timeslips.org/) stresses the power of imagination; Gerry Glazner’s *Alzheimer’s Poetry project* (http://www.alzpoetry.com/index.php/) centers on the emotional force of poetry (Swinnen 2014).

10 Fien is perfectly aware that her performance exceeds many boundaries, as evidenced by her remark that she is cleanliness itself. Her idea of cleanliness reveals a deep seated cultural reflex, which has been described by anthropologist Mary Douglas and which cannot be ignored without cost: our inclination towards purity as line of defense against external dangers in the form of dirt, disease, and contagion. The increasing blurring of the boundaries of our existence seems to confirm our biggest fear pertaining to dementia—the fear of losing our self.

11 Kamphof here relies on work by Heidegger (*The Age of the Worldview*), Merleau-Ponty (*Phenomenology of Perception*), and Nietzsche (*On the Use and Abuse of History for Life*).

12 This also explains the dominance of calls for evidence-based observational research in discussions on the value of art in health- and dementia care (for critiques, see: de Medeiros and Basting 2013; Oliver 2009; Raw et al. 2012). In line with the evidence-based paradigm of medical and psychological disciplines, existing studies of clowning in care for the elderly generally focus on the observed effectiveness of what is called ‘humor and laughing therapy’ (e.g. Spitzer 2011; Warren and Spitzer 2011). Moreover, the miMakkus foundation itself also builds on the promise of observational (e.g. video-based) research (cf. Wintels et al. 2014).

13 Cf. Stichting miMakkus (2015).

14 Such focus on the person in the here and now by the clown who tries to be “fully in the present moment” (de Graan 2012: 69) can be defended on philosophical grounds as well. Rebecca Dresser (1995), a critic of Dworkin, claims that the psychological discontinuity on account of dementia can be so large that the interests and preferences of the earlier person do not constitute a good standard in care. She argues for starting from the person in his/her current condition.

15 Kamphof bases this alternative history of the gaze on Baudrillard (*Simulacra and Simulation*), Frazer’s history of magic (*The Golden Bough*), Freedberg (*The Power of Images*), Lyotard (*The Inhuman*), Merleau-Ponty (*Phenomenology of Perception*), Sobchack (*Carnal Thoughts*), and others.

16 Unless indicated otherwise, I quote in this section from field notes recorded during my miMakkus training, including internships (March 2008–2009), and from my observations in Saint Anna involving Fien/Tessa Brouwer (February–March 2011).

17 The shift in this quotation of the first-person perspective from the clown—‘I as clown’—to that of the resident—‘for me as resident’—and further to a multiple ‘we’ perhaps occurs unknowingly, but it well mirrors what Rauh has in mind when it comes to adopting the disposition or stance of the resident.

18 In their recent study based on video-ethnography of elder-clowning, Kontos et al. (2015: 13) further elaborate the reciprocal nature of clown-resident engagement. They show in empirically rich detail how residents actively contribute in both verbal and embodied ways to so-called “relational presence” that emerges in “affective relationality, reciprocal playfulness, and co-constructed imagination.”

19 I follow here my analysis of reciprocal affect (Hendriks 2012), based on work of Despret (2004) and Latour (2004) that I present as critical supplement to inspiring work of Kontos (2005) on the role of the body in dementia care. My argument is that clown and resident become equally engaged in sensitizing the other to their own bodily ways and language, without which they would remain grey and indifferent to each other. The image that comes to mind in the latter case is a play in which two actors both, at the same time, are playing the part of sleeping beauty as well as the prince (cf. Killick and Allan 2012), but never manage to meet each other. In order for both clown and resident to become expressive and articulate in a way that *matters* to the other we thus need to complement an understanding of expressive capacities of the subject as “intrinsic” (Kontos et al. 2015: 13), with an understanding that values the creativity and artifice that goes into *acquiring* a body, i.e. learning to become sensitized to and affected by subtle ways of the other.

20 Cf. factors listed in the system of Dementia Care Mapping (Brooker and Surr 2005) as undermining personhood. I have a more specific type of factors in mind, however, that undermine the possibility to acquire a sensitive body and to learn to see again.

21 Interview miMakkus-clown instructor Titia Brassé, Geleen, 10 April 2015.

## References

[CR1] Basting Anne Davis (2009). Forget memory: Creating better lives for people with dementia.

[CR2] Brooker Dawn, Surr Claire (2005). Dementia care mapping: Principles and practice.

[CR3] Despret Vinciane (2004). The body we care for: Figures of anthropo-zoo-genesis. Body and Society.

[CR4] Dresser Rebecca (1995). Dworkin on dementia: Elegant theory, questionable policy. Hastings Center Report.

[CR5] Dworkin Ronald (1993). Life’s dominion: An argument about abortion, euthanasia, and individual freedom.

[CR6] de Graan, Roland. 2012 A fool’s view on transformation. Unpublished master thesis, Utrecht: Utrecht School of Governance, Utrecht University.

[CR22] de Medeiros Kate, Basting Anne (2013). “Shall I compare thee to a dose of Donepezil?”: Cultural arts interventions in dementia care research. The Gerontologist.

[CR7] Griffin Randy (2012). Changing the culture for dementia care.

[CR8] Hendriks Ruud (2012). Tackling indifference: Clowning, dementia, and the articulation of a sensitive body. Medical Anthropology: Cross-Cultural Studies in Health and Illness.

[CR9] Hertogh Cees, de Boer Marike, Dröes Rose-Marie, Eefsting Jan (2007). Would we rather lose our life than lose our self? Lessons from the Dutch debate on euthanasia for patients with dementia. The American Journal of Bioethics.

[CR10] Hughes Julian, Louw Stephen, Sabat Steven, Hughes Julian, Louw Stephen, Sabat Steven (2006). Seeing whole. Dementia. Mind, meaning and the person.

[CR11] Jaworska Agniezka (1999). Respecting the margins of agency: Alzheimer’s patients and the capacity to value. Philosophy and Public Affairs.

[CR12] Kamphof Ike (2012). Iedereen voyeur. Kijken en bekeken worden in de 21ste eeuw.

[CR13] Killick John, Allan Kate (2012). Playfulness and dementia: A practical guide.

[CR14] Kitwood Tom (1990). The dialectics of dementia: With particular reference to Alzheimer’s disease. Ageing and Society.

[CR15] Kitwood Tom (1997). Dementia reconsidered. The person comes first.

[CR16] Kitwood Tom, Benson Sue (2004). The new culture of dementia care.

[CR17] Kontos Pia (2005). Embodied selfhood in Alzheimer’s disease: Rethinking person-centred care. Dementia.

[CR18] Kontos Pia, Miller Karen-Lee, Mitchell Gail, Stirling-Twist Jan (2015). Presence redefined: The reciprocal nature of engagement between elder-clowns and persons with dementia. Dementia.

[CR19] Koppelman Elysa (2002). Dementia and dignity: Towards a new method of surrogate decision making. The Journal of Medicine and Philosophy.

[CR20] Latour Bruno (2004). How to talk about a body? The normative dimension of science studies. Body and Society.

[CR21] Lee Hilary, Adams Trevor (2011). Creative approaches in dementia care.

[CR23] Mol Annemarie, Moser Ingunn, Pols Jeanette, Mol Annemarie, Moser Ingunn, Pols Jeanette (2010). Care: Putting practice into theory. Care in practice: On tinkering in clinics, homes and farms.

[CR24] Oliver James (2009). Creativity as openness: Improvising health and care ‘situations’. Health Care Analysis.

[CR25] Pols Jeanette (2005). Enacting appreciations: Beyond the patient perspective. Health Care Analysis.

[CR26] Rauh Jan (2013). Persoonlijke aantekeningen.

[CR27] Raviv Ammon (2013). Humor in the ‘Twilight Zone’: My work as a medical clown with patients with dementia. Journal of Holistic Nursing.

[CR28] Raw Anni, Lewis Susan, Russell Andrew, Macnaughton Jane (2012). A hole in the heart: Confronting the drive for evidence-based impact research in arts and health. Arts and Health.

[CR29] Robb David (2007). Clowns, fools and picaro’s. Popular forms in theatre, fiction and film.

[CR30] Schermer Maartje (2003). Dementie en het levensverhaal; de balans tussen verleden en heden. Ethiek and Maatschappij.

[CR31] Schermer Maartje (2007). Nothing but the truth? On truth and deception in dementia care. Bioethics.

[CR32] Simonds Caroline, Warren Bernie (2004). The clown-doctor chronicles.

[CR33] Spitzer Peter, Lee Hilary, Adams Trevor (2011). LaughterBoss™. Creative approaches in dementia care.

[CR34] Stichting miMakkus. 2015. *Rapportage na miMakkerbezoek*. Eindhoven: Stichting miMakkus. http://www.mimakker.com/rapportage-na-mimakkerbezoek/.

[CR35] Swinnen Aagje (2014). Healing words: A study of poetry interventions in dementia care. Dementia.

[CR36] Symons, David. 2012 A study of elderclown programs in Scotland, the Netherlands, USA and Canada. Retrieved from https://www.churchilltrust.com.au/media/fellows/2012_Symons_David.pdf.

[CR37] Taussig Michael (1993). Mimesis and alterity. A particular history of the senses.

[CR38] Taylor Janelle (2008). On recognition, caring, and dementia. Medical Anthropology Quarterly.

[CR39] Tronto Joan (1993). Moral boundaries: A political argument for an ethic of care.

[CR40] Warren Bernie, Warren Bernie (2008). Healing laughter: The role and benefits of clown-doctors working in hospitals and healthcare. Using the creative arts in healthcare and therapy.

[CR41] Warren Bernie, Spitzer Peter (2011). Laughing to longevity—The work of elder clowns. The Lancet.

[CR42] Winance Myriam, Mol Annemarie, Moser Ingunn, Pols Jeanette (2010). Care and disability: Practices of experimenting, tinkering with, and arranging people and technical aids. Care in practice: On tinkering in clinics, homes and farms.

[CR43] Wintels Sophie, van Oorsouw Wietske, Hendriks Ruud, Embregts Petri (2014). miMakkus: Belevingsgerichte zorg met behulp van clownerie voor mensen met een ernstige verstandelijke beperking.

